# Cytotoxicity of Co(III) Complexes of Arginine

**DOI:** 10.1155/MBD.2001.195

**Published:** 2001

**Authors:** Petia Genova, Tatiana Varadinova, Radostina Alexandrova, Sreco Trifunovic, Petar Radivojsa

**Affiliations:** 1 Laboratory of Virology Faculty of Biology Sofia University 8 Dragan Tzankov Blvd. Sofia 1421 Bulgaria; 2 Institute of Experimental Pathology and Parasitology Bulgarian Academy of Sciences b1.25, Georgy Bonchev Blvd Sofia 1113 Bulgaria; 3 Department of Chemistry Faculty of Science University of Kragujevac 12 R. Domanovica Kragujevac 3400 Serbia and Montenegro; 4 Faculty of Chemistry Universityof Belgrade Studentski trg.16 P.O.Box 158, Belgrade 11000 Serbia and Montenegro

## Abstract

The cytotoxicity of four Co(III) complexes of arginine on nontumour MDBK cells and on two cell lines derived from transplantable tumors, LSCC-SF(Mc29) and LSR-SF (SR), was evaluated comparative!y. Based on the cytotoxic concentration required to inhibit cell surveillance by 50% cc_∇'_ it was found that: (i) the cytotoxicity of complexes tested increases when the concentration decreased; (ii) the cell surveillance depends on both complex and cell specificities. The complex specificity was illustrated by the order **1** > **4** > **2** ≥ **3** . The cell specific response was demonstrated by the fact that LSCC-SG (Mc29) cells were up to 60 times more sensitive to **1** while LSR-SF (SR) cells were up to 1000 times more sensitive to **2** as compared to MDBK cells. Furthermore, with the prolongation of action on nontumour cells the cytotoxicity of **4** decreased up to 300 times while for both tumour cells it was independent on the duration of action.


### CYTOTOXICITY OF Co(III) COMPLEXES OF ARGININE

Petia Genova[1], Tatiana Varadinova*[1], Radostina Alexandrova[2], Sreco Trifunovic[3], and Petar Radivojsa[4]

[1]Laboratory of Virology, Faculty of Biology, Sofia University, 8 Dragan Tzankov Blvd., 1421 Sofia, Bulgaria <t_varadinova@yahoo.com>
[2]Institute of Experimental Pathology and Parasitology, Bulgarian Academy of Sciences, bl.25, Georgy Bonchev Blvd, 1113 Sofia, Bulgaria
[3]Department of Chemistry, Faculty of Science, University of Kragujevac, 12 R. Domanovica, 3400 Kragujevac, Yugoslavia
[4]Faculty of Chemistry, University of Belgrade, Studentski trg.16, P.O.Box 158, 11000 Belgrade, Yugoslavia

**ABSTRACT**

The cytotoxicity of four Co(III) complexes of arginine on nontumour MDBK cells and on two cell lines derived from transplantable tumors, LSCC-SF(Mc29) and LSR-SF (SR), was evaluated comparative'y. Based on the cytotoxic concentration required to inhibit cell surveillance by 50% ($CC_{50}$) it was found that: (i) the cytotoxicity of complexes tested increases when the concentration decreased; (ii) the cell surveillance depends on both complex and cell specificities. The complex specificity was illustrated by the order **1** > **4** > **2** ≥ **3**. The cell specific response was demonstrated by the fact that LSCC-SG (Mc29) cells were up to 60 times more sensitive to **1** while LSR-SF (SR) cells were up to 1000 times more sensitive to **2** as compared to MDBK cells. Furthermore, with the prolongation of action on nontumour cells the cytotoxicity of **4** decreased up to 300 times while for both tumour cells it was independent on the duration of action.

**Key words:** cytotoxicity, MDBK, LSR-SF(SR), LSCC-SF(Mc29), cobalt(III), arginine.

### INTRODUCTION

Being essential metal cobalt influences different physiological and enzymatic functions. Participating in the corrin ring of vitamin $B_{12}$, cobalt plays a crucial role in a number of biological functions [1]. Furthermore, cobalt speeds up ATP turnover [2], activates arginase and inhibits δ-aminolevulinic acid synthethase [3], affects liver mixed-function oxidase [4], enhances acylamino acid hydrolase [5] and yeast elonase [6]. Eight enzymes have been identified in which cobalt is in a form other than that in the corrin ring [7].

The antitumour activity of cobalt compounds has also been reported. Thus, several alkyne-cobalt carbonyl compounds inhibit the growth of human melanoma and carcinoma cells [8]. Moreover, organocobalt(III) compounds potentate the antineoplastic effect of conventional agents such as cis-DDP, radiation and local hyperthermia [9]. We reported [10] that the cytotoxicity of Co(II) complexes of aminoacids is predetermined by the specificities of the particular ligand. Thus, complexes of essential aminoacids, such as lysine, arginine and histidine were 10 times less toxic than the complex of serine.

However, up to now there are no published data showing that the response against cobalt complexes is predetermined by the specificities of the particular cells. This is why the aim of the present study was to evaluate comparatively the cytotoxicity of four Co(III) complexes with arginine on two tumour cell lines derived from transplantable tumors and on one nontumour.

### MATERIALS AND METHODS

The investigated complexes are syntethised according to the procedures published earlier for the complexes **1**, **2** and **4** [11] and for the complex **3** [12].

Table 1. Co(III) complexes of arginine

| Nr | Complex | Mw |
|---|---|---|
| **1** | L-/+/$_D$-*mer*-[Co(S-argH)$_3$](NO$_3$)$_3$2H$_2$O | 587.54 |
| **2** | D-/+/$_D$-*fac*-[Co(S-argH)$_3$](NO$_3$)3H$_2$0 | 632.55 |
| **3** | D-(-)$_D$-*cis*(NO$_2$)-*trans*(N)-[Co(S-argH)$_2$(NO$_2$)$_2$]Cl0.5H$_2$O | 532.83 |
| **4** | (-)$_D$-*anti*(N)-D-*cis*(N),*cis*(O)-L-*cis*(N), *cis*(O)-[Co$_2$(s-argH)$_4$(OH)$_2$]Cl$_4$.4H$_2$0 | 1058.48 |

***Cells.*** Three cell lines were used in the experiments. The nontumour one was derived from bovine kidney cells, MDBK. The other two were derived from transplantable tumor in rat induced by Rous sarcoma virus, strain Schmidt- Ruppin, LSR-SF(SR) and in chicken induced by myeolocytomatosis Mc29 virus, LSC-SF(Mc29). Cells from all three lines were grown at 37°C in RPMI-1640 medium (GIBCO BRL) supplemented with 10% bovine serum (BS) and antibiotics. During the experiments the medium was supplemented with 5% BS.

***Methods of detecting the effect on cell viability, concentration required to inhibit cell viability by 50% ($CC_{50}$) and maximal nontoxic concentration (MNC).*** Co(III) complexes were first dissolved in DMSO till a concentration of 1M was obtained. Dilutions were made in cell growth medium. Cells were seeded into 96 well tissue culture plates at a concentration of $1x10^4$ cells/ml and cultured at 37°C in $CO_2$ atmosphere. Confluent monolayers were washed and covered with media modified with the appropriate conpound in ten-fold dilutions starting from 10mM till 0.1μM. Cytopathic effects were read on the 24h and 48h after culturing cells at 37°C by microscopy of unstained monolayers and by trypan blue exclusion test. The cell viability was calculated as a percent from the total number of cells per sample. The dose-response relationships were constructed by linearly regressing drug concentrations against the percent inhibition of viability values for the cell control. The $CC_{50}$ of the each compound was calculated from dose-response curves. Each experiment was done in duplicate.

### RESULTS AND DISCUSSION

During experimentations it was found that the cytotoxicity of all four Co(III) complexes of arginine increased when the concentration decreased (Fig. 1). Moreover, this phenomenon was independent on both complex and cell specificities.

Fig. 1. Dynamics of cell surveillance under the influence of Co(III) complexes of arginine.

A. MDBK cells

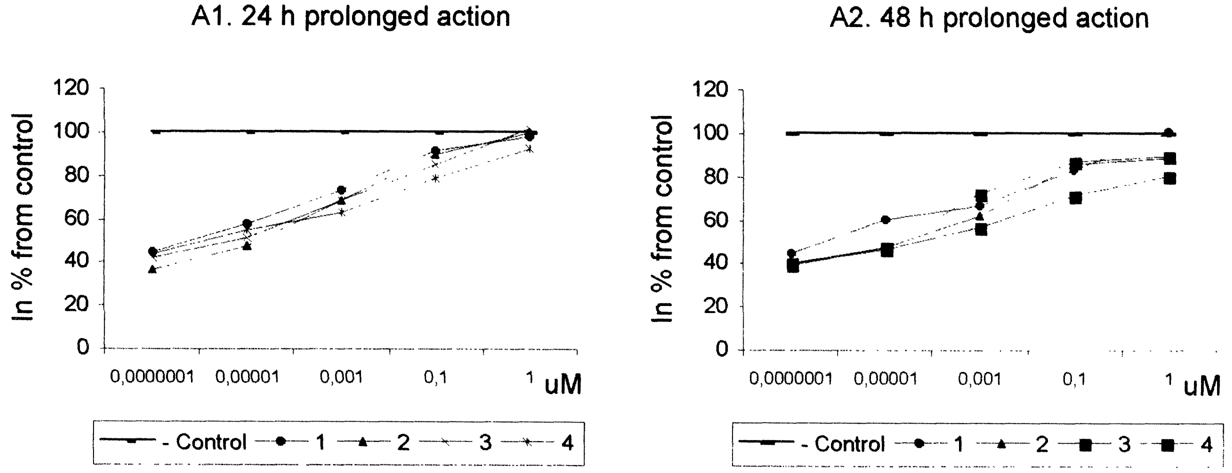

B. LSCC-SF(Mc29)

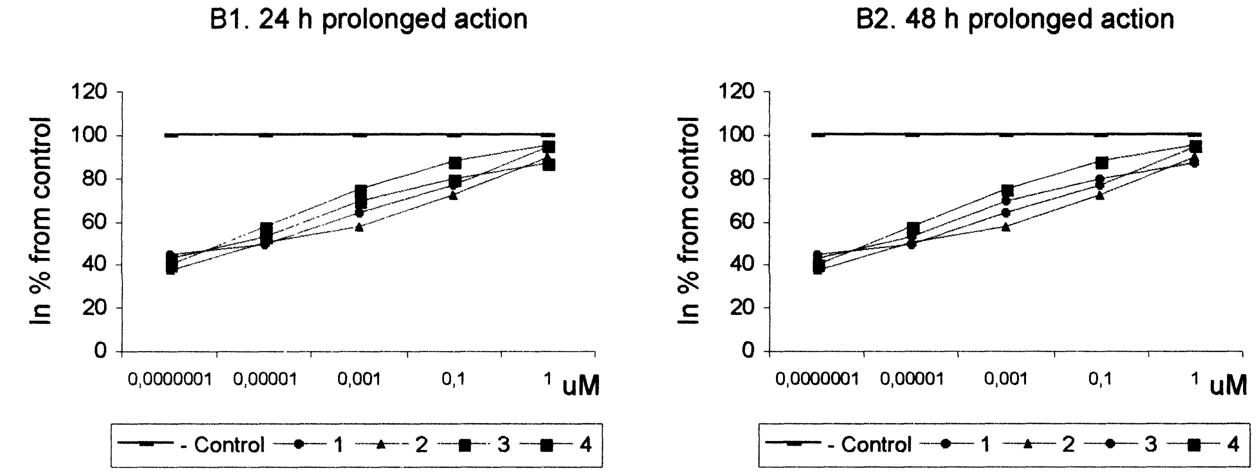

C. LSR-SF(SR) cell

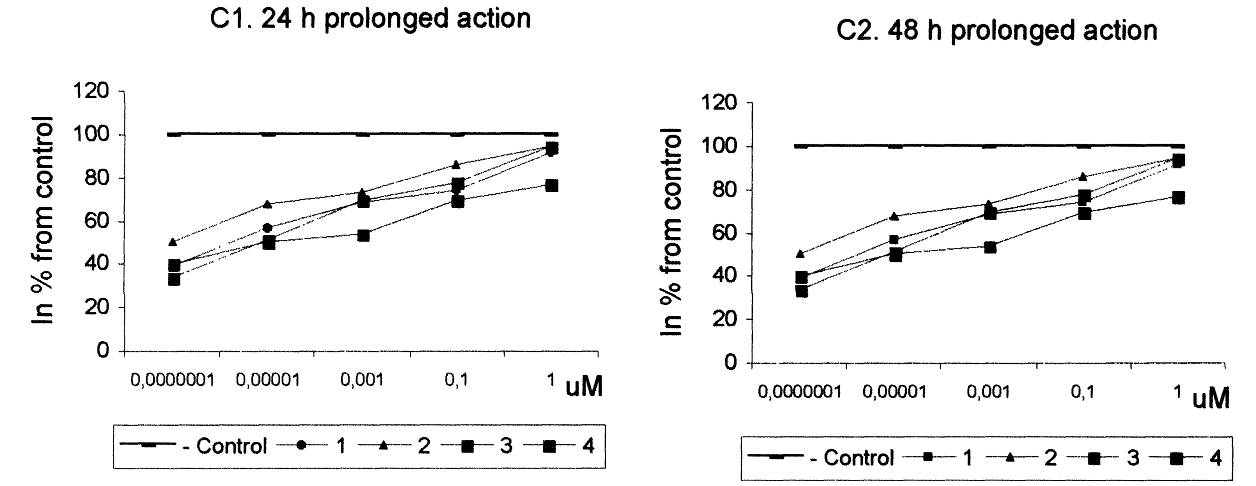

On the contrary, based on the data from $CC_{50}$ it was found that the cell surveillance depended on both complex and cell specificities (table 2). Thus, complex specificities were manifested by the fact that **1** was the most cytotoxic complex out of for complexes tested. This could be due to a specific geometrical and absolute configuration of **1** that causes different ways of dissociation in the inner sphere of the complexes. Also, the free guanidine groups of the arginine give the possibility to the complex for easy interactions with different biomolecules in the cell forming hydrogen bonds and other dipol-dipol interactions. In addition, the highest cytotoxicity of **1** could be also due to $NO_3^-$ ions participating in the outer sphere, as it is known that the activity of anions decreases in the order $NO_3^-$ > Cl > $NO_2^-$. This could be also the reason of the increased cytotoxicity of **2** for tumour but not for nontumour cells.

Table 2. $CC_{50}$ values of Co(III) complexes of arginine*

| Complex | MDBK | | LSR-SF(SR) | | LSCC-SF(Mc29) | |
|---|---|---|---|---|---|---|
| | 24h♦ | 48h♦ | 24h | 48h | 24h | 48h |
| **1** | 0.0001 | 0.001 | 0.004 | 0.008 | 0.0001 | 0.0006 |
| **2** | 0.1 | 0.1 | 0.0001 | 0.002 | 0.01 | 0.04 |
| **3** | 0.01 | 0.1 | 0.1 | 0.7 | 0.01 | 0.1 |
| **4** | 0.001 | 0.3 | 0.01 | 0.01 | 0.003 | 0.001 |

* - data in μM; ♦ - duration of action

The cell specific response was deduced from the following data: (i) LSCC-SF(Mc29) cells were up to 60 times more sensitive to **1** while LSR-SF(SR) cells were up to 1000 tomes more sensitive to **2** as compared to nontumour MDBK cells, (ii) LSCC-SF(Mc29) cells were up to 30 times more sensitive to **4** than LSR-SF(SR) and MDBK cell and, (iii) with the prolongation of action on MDBK cells the cytotoxicity of **4** decreased up to 300 times while in tumour cells it was independent on the duration of action. These data show that the increased cytotoxicity of **1**, **2** and **3** for actively divided and nondifferentiated tumour cells as compared to that for nontumour cells could also be due to the inhibition of genome *trans*-activation as was shown by Louie and Meade [13] for cobalt compounds.

However, the effect of **3** was: (i) independent on cell specificities as was demonstrated by $CC_{50}$ values following the same range in all three cell lines independently on their specificities and, (ii) decreased in parallel with the duration of treatment.

**ACKNOWLEDGEMENT**

The authors are grateful for the financial support by the Ministry of Science and Technology of the Republic of Serbia.

**REFERENCES**

1. J. Anger , R. Heinrich, *Handbook of Toxicity of Inorganic Compounds*, **20**, 251 (1988)

2. G. R. Meyer, R.Cornelius, *J. Inorg.Biochem.*, **22**, 249 (1984)

3. N.Schoenfeld, Y.Greenblat, O.Epstein, D.P.Tschundy, A.Atsmon, *Biochem.Pharmacol.*, **32**, 2333 (1983)

4. D.A.Schoeller, A.N.Kotake, G.H.Lambert, P.S.Krager, A.L.Baker, *Hepatology*, **5**, .276 (1985)

5. J.Gilles, H.G.Loffler, F.Schneider, Z.Naturforsch, **c39**, 1017 (1984)

6. S.L.Rose, L.Ch.Dickinson, E.W.Westhead, *J.Biol.Chem.*, **259,** 4405 (1984)

7. J.J.R.F.Da Silva, *The Inorganic Chemistry of Life*, 1989

8. R. Lauwerys,D.Lison, *Sci. Total Environ.*, **30**, 150 (1994)

9. D. Cartlen, *NIOSH Registry of Toxic Effects of Chemical Substances*, 276 (1976)

10. M.Kobayashi, S.Shimizu. *Eur.J.Biochem*, **4**, 261 (1999)

11. P.N.Radivojsa, N.Juranic, M.B.Celap, K.Tariumi and K.Saito, *Polyhedreon,* **10,** 2717 (1991)

12. M.B.Celap, M.J.Malinar, P.N.Radivojsa and Lj.Solujic, *Inorg. Synth.*, **23,** 91 (1985)

**Received: December 10, 2001 – Accepted: December 13, 2001 – Accepted in publishable format: December 17, 2001**

